# A role for placental kisspeptin in **β** cell adaptation to pregnancy

**DOI:** 10.1172/jci.insight.124540

**Published:** 2019-10-17

**Authors:** James E. Bowe, Thomas G. Hill, Katharine F. Hunt, Lorna I.F. Smith, Sian J.S. Simpson, Stephanie A. Amiel, Peter M. Jones

**Affiliations:** School of Life Course Sciences, King’s College London, London, United Kingdom.

**Keywords:** Endocrinology, Reproductive Biology, Diabetes

## Abstract

During pregnancy the maternal pancreatic islets of Langerhans undergo adaptive changes to compensate for gestational insulin resistance. Kisspeptin has been shown to stimulate insulin release, through its receptor, GPR54. The placenta releases high levels of kisspeptin into the maternal circulation, suggesting a role in modulating the islet adaptation to pregnancy. In the present study we show that pharmacological blockade of endogenous kisspeptin in pregnant mice resulted in impaired glucose homeostasis. This glucose intolerance was due to a reduced insulin response to glucose as opposed to any effect on insulin sensitivity. A β cell–specific GPR54-knockdown mouse line was found to exhibit glucose intolerance during pregnancy, with no phenotype observed outside of pregnancy. Furthermore, in pregnant women circulating kisspeptin levels significantly correlated with insulin responses to oral glucose challenge and were significantly lower in women with gestational diabetes (GDM) compared with those without GDM. Thus, kisspeptin represents a placental signal that plays a physiological role in the islet adaptation to pregnancy, maintaining maternal glucose homeostasis by acting through the β cell GPR54 receptor. Our data suggest reduced placental kisspeptin production, with consequent impaired kisspeptin-dependent β cell compensation, may be a factor in the development of GDM in humans.

## Abstract 2

Placental kisspeptin regulates islet adaptation to pregnancy that is necessary for preventing gestational diabetes in mice and humans.

## Introduction

Kisspeptins are a family of peptides, encoded by the KISS1 gene, which are the endogenous ligands for the Gq-protein–coupled receptor GPR54, also commonly known as KISS1R. Kisspeptin is well known for its permissive role acting locally in the hypothalamus to regulate puberty and reproductive function (1, 2), but its role in other physiological systems is much less understood. GPR54 is highly expressed in several peripheral tissues (3), including insulin-secreting pancreatic β cells (4), consistent with a role for kisspeptin in regulating glucose homeostasis (4). However, there are discrepancies in the literature about the effects of exogenous kisspeptin on β cell function. We have previously reported that exogenous kisspeptin enhances glucose-induced insulin secretion from rodent, porcine, and human islets in vitro (5, 6), and in rats and mice in vivo (7), consistent with other reports of stimulatory effects in vitro (8) and in vivo in non-human primates (9) and in humans (10). However, others report predominantly inhibitory effects in vitro (11, 12). Similarly, studies investigating the physiological role(s) of endogenous kisspeptin in glucose homeostasis are seemingly contradictory. Thus, it has been suggested that low levels of hepatic kisspeptin are released to impair β cell function and play a role in the development of type 2 diabetes (13), while GPR54-null mice become glucose intolerant, consistent with stimulatory effects of kisspeptin on β cell function (14, 15). Aspects of the metabolic phenotype may also be sex specific because female GPR54-null mice develop increased adiposity and impaired glucose tolerance that are not observed in males (16).

Circulating kisspeptin levels are extremely low under most physiological circumstances. The exception is in pregnancy, when kisspeptin is released from the placenta into the maternal circulation such that circulating levels increase several thousand–fold in humans (17). The circulating kisspeptin during pregnancy in animal models is unclear because of the lack of reliable assays; however, kisspeptin mRNA is expressed in rodent placenta and increases through pregnancy (18–20). Thus, one potential physiological role for β cell GPR54 is in regulating β cell adaptive responses to pregnancy. Pregnancy presents the maternal metabolism with the problem of providing for the energy requirements of the growing fetus while maintaining fuel homeostasis in the mother. In both rodents and humans, there are reversible adaptive changes, including a progressive increase in maternal insulin resistance, which, under normal circumstances, is countered by increased maternal insulin secretion to maintain normoglycemia. The increased insulin secretory capacity is provided through both functional and structural changes to the islets (21). Failure of these compensatory mechanisms may result in the development of glucose intolerance in pregnancy, known as gestational diabetes mellitus (GDM) in humans (22). At present our understanding of the signals regulating β cell adaptations to pregnancy is incomplete. Previous studies have demonstrated a role for prolactin and placental lactogens (23, 24), but lactogenic hormones alone are unlikely to account for all the β cell adaptive responses. The timing of their increased secretion does not mirror that of the adaptive responses, and there is interest in identifying other contributing signals. Placental production of kisspeptin, and the consequent increased level of kisspeptin in the maternal circulation, closely reflect the time course of the increased insulin secretory capacity of maternal β cells (25, 26).

We here demonstrate that placental kisspeptin acts as an important signal to amplify insulin secretory responses as needed to compensate for increased insulin resistance during pregnancy. We show that, in experimental animals, pharmacological blockade or genetic ablation of β cell GPR54 during pregnancy results in a failure of normal homeostatic mechanisms and the consequent development of maternal glucose intolerance. Furthermore, we demonstrate that low circulating levels of kisspeptin during human pregnancy are associated with GDM. Therefore, placental kisspeptin acting via β cell GPR54 is essential for normal glucose homeostasis during pregnancy.

## Results

### Effects of pharmacological activation or blockade of GPR54 on glucose homeostasis.

**Figure 1:**
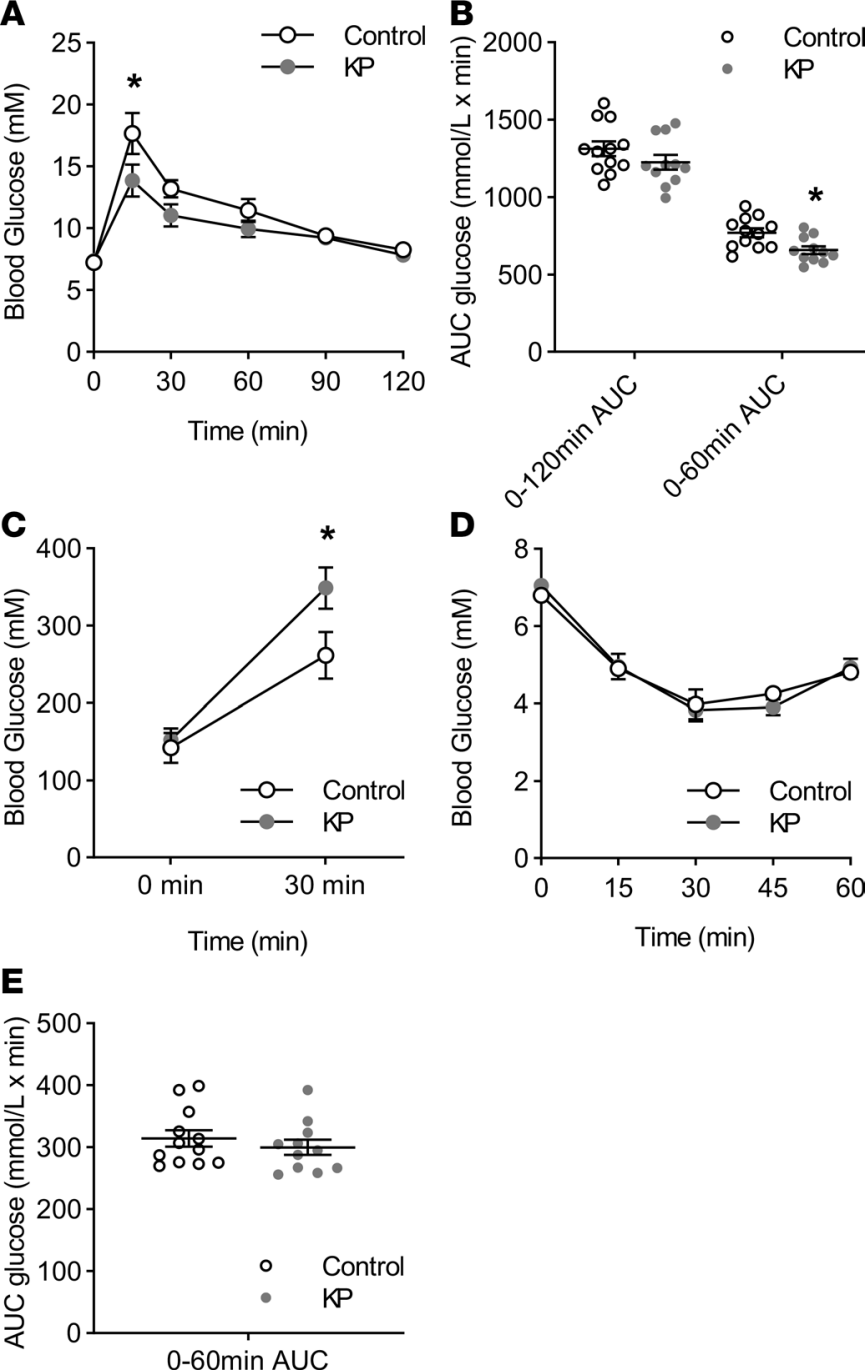
In vivo effects of chronic kisspeptin administration on glucose homeostasis in nonpregnant mice. (**A**) In nonpregnant female ICR mice, chronic subcutaneous administration of kisspeptin resulted in improved glucose tolerance after intraperitoneal (i.p.) glucose administration (2 g/kg) when compared with untreated controls (2-way repeated-measures ANOVA, 15 minutes *P* < 0.001). (**B**) There was no significant difference in overall 120 minutes area under the curve (AUC), but kisspeptin treatment did result in a significant reduction in glucose AUC over 0–60 minutes (2-tailed Student’s *t* test, *P* = 0.0321). (**C**) Although chronic kisspeptin treatment had no significant effect on fasting plasma insulin levels, kisspeptin-treated animals had significantly increased insulin release in response to i.p. glucose administration (2  g/kg) after 30  minutes when compared with controls (1-way ANOVA, *P* = 0.033). (**D**) Kisspeptin had no effect on the plasma glucose response to i.p. insulin administration (0.75  IU/kg) when compared to controls, through comparison of both individual time points and (**E**) glucose AUC. Mean ± SEM, *n* = 11–12, and *P < 0.05 vs. control.

Chronic activation of GPR54 improved glucose tolerance and insulin secretion in mice, consistent with a role for circulating kisspeptin in glucose homeostasis. Thus, kisspeptin administration to nonpregnant mice for 8 days resulted in a significant improvement in glucose tolerance as assessed by an intraperitoneal glucose tolerance test, with significant reductions in blood glucose at 15–30 minutes after administration (Figure 1A). However, this was not reflected in the overall area under the curve (AUC) data (Figure 1B) because blood glucose levels were similar in both treatment groups from 60 to 120 minutes. The difference in glucose profile was associated with significantly elevated plasma insulin levels in kisspeptin-treated mice 30 minutes after glucose administration, while basal insulin levels were not altered (Figure 1C). There were no significant differences in plasma glucose between treatment groups in an insulin tolerance test (Figure 1, D and E). Thus, circulating kisspeptin enhances β cell secretory responses to glucose challenge.

**Figure 2:**
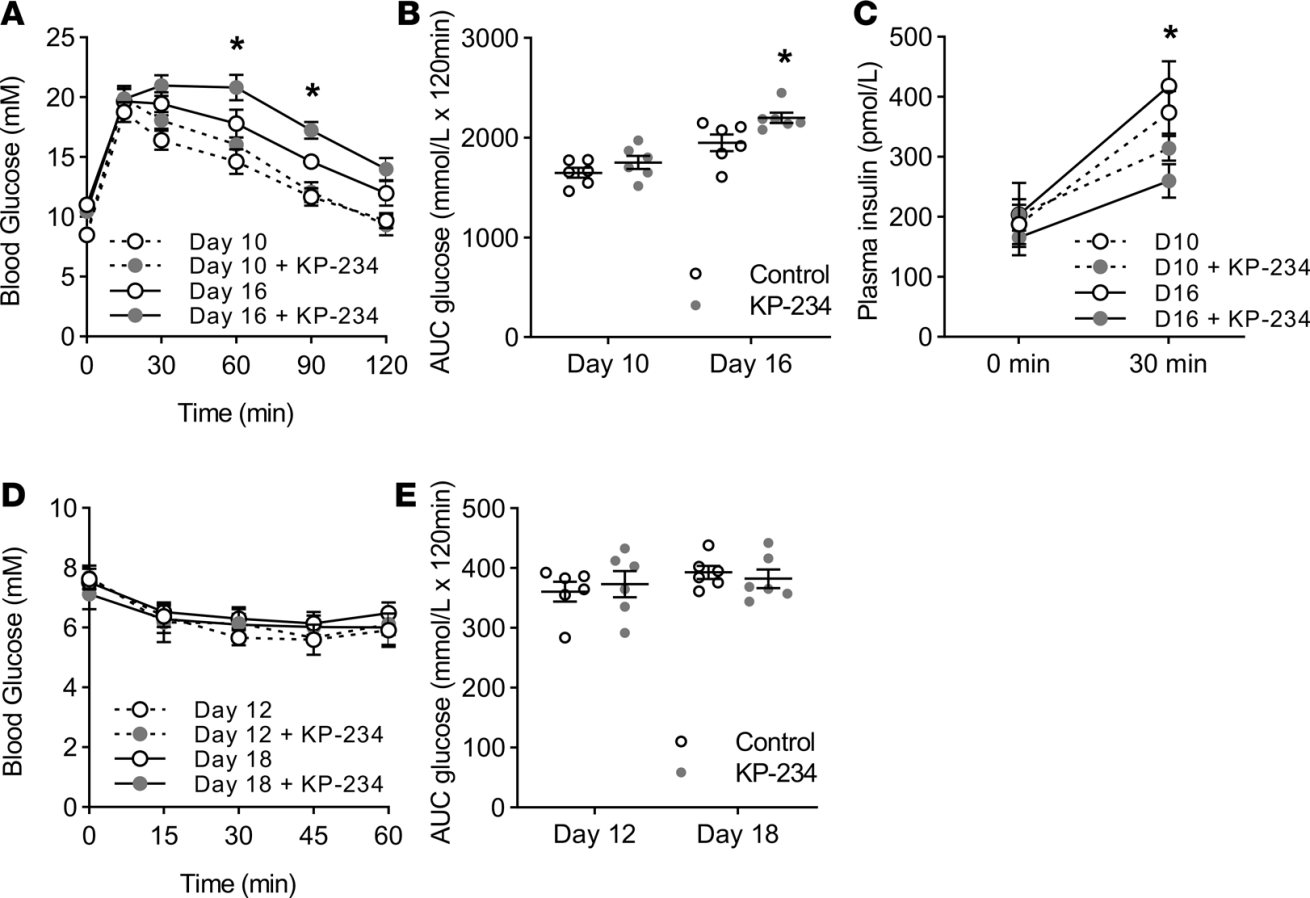
Effects of kisspeptin-234 on glucose homeostasis during pregnancy in mice. (**A**) At day 10 of pregnancy chronic kisspeptin-234 (KP-234) administration had no significant effect on glucose tolerance; however, at day 16 of pregnancy mice treated with KP-234 had significantly impaired glucose tolerance following i.p. glucose administration (2  g/kg) when compared with untreated pregnant controls, as determined by both comparison of individual time points (2-way repeated-measures ANOVA; 60 minutes *P* = 0.022; 90 minutes *P* = 0.046) and (**B**) glucose AUC over the course of the test (1-way ANOVA, *P* = 0.031). (**C**) Blocking endogenous kisspeptin signaling with KP-234 did not have any effect on fasting plasma insulin levels at either day 10 or day 16 of pregnancy. At day 16 chronic KP-234 treatment resulted in significantly reduced insulin release in response to i.p. glucose administration (2  g/kg) after 30  minutes when compared with pregnant controls (1-way ANOVA, *P* = 0.009). A similar trend was observed at day 10; however, this was not significant. (**D**) KP-234 had no effect on the plasma glucose response to i.p. insulin administration (0.75  IU/kg) when compared to untreated controls at either day 12 or day 18 or pregnancy, through both comparison of individual time points and (**E**) glucose AUC. Mean ± SEM; *n* = 6; *P < 0.05 vs. control.

Pharmacological blockade of GPR54 caused impaired insulin secretion and impaired glucose tolerance in pregnant mice, as shown in Figure 2. Pregnancy in ICR mice is associated with impaired glucose tolerance and increased insulin resistance at both days 10–12 and days 16–18 of pregnancy when compared with nonpregnant controls (Supplemental Figure 1, A–C; supplemental material available online with this article; https://doi.org/10.1172/jci.insight.124540DS1), and this was associated with enhanced glucose-induced insulin secretion, as assessed by significantly higher plasma insulin levels in the pregnant mice 30 minutes after a glucose load (Figure 2B). Chronic administration of the GPR54 antagonist kisspeptin-234 to pregnant mice further impaired glucose tolerance by day 18 of pregnancy (Figure 2, A and B), which was associated with significant reductions in glucose-stimulated insulin secretion (Figure 2C) but without any effect on basal plasma insulin levels (Figure 2C). Blocking endogenous kisspeptin by antagonist administration had no detectable effects on the pregnancy-dependent increases in insulin resistance observed at either mid-pregnancy (day 12) or late pregnancy (day 18) (Figure 2, D and E). Thus, blocking activation of GPR54 by endogenous kisspeptin during pregnancy induces glucose intolerance by reducing glucose-induced insulin secretion rather than through effects on insulin target tissues.

### Effects of β cell GPR54 deletion on in vitro islet function.

To assess whether the metabolic effects of pharmacological blockade of GPR54 were mediated primarily through β cells, we generated a β cell–specific GPR54-knockout mouse model (β cell GPR54^–/–^). Successful β cell–specific knockdown of GPR54 was confirmed and the model validated (Supplemental Figures 2 and 3). Before in vivo experiments islets from β cell GPR54^–/–^ mice were isolated for in vitro characterization.

**Figure 3:**
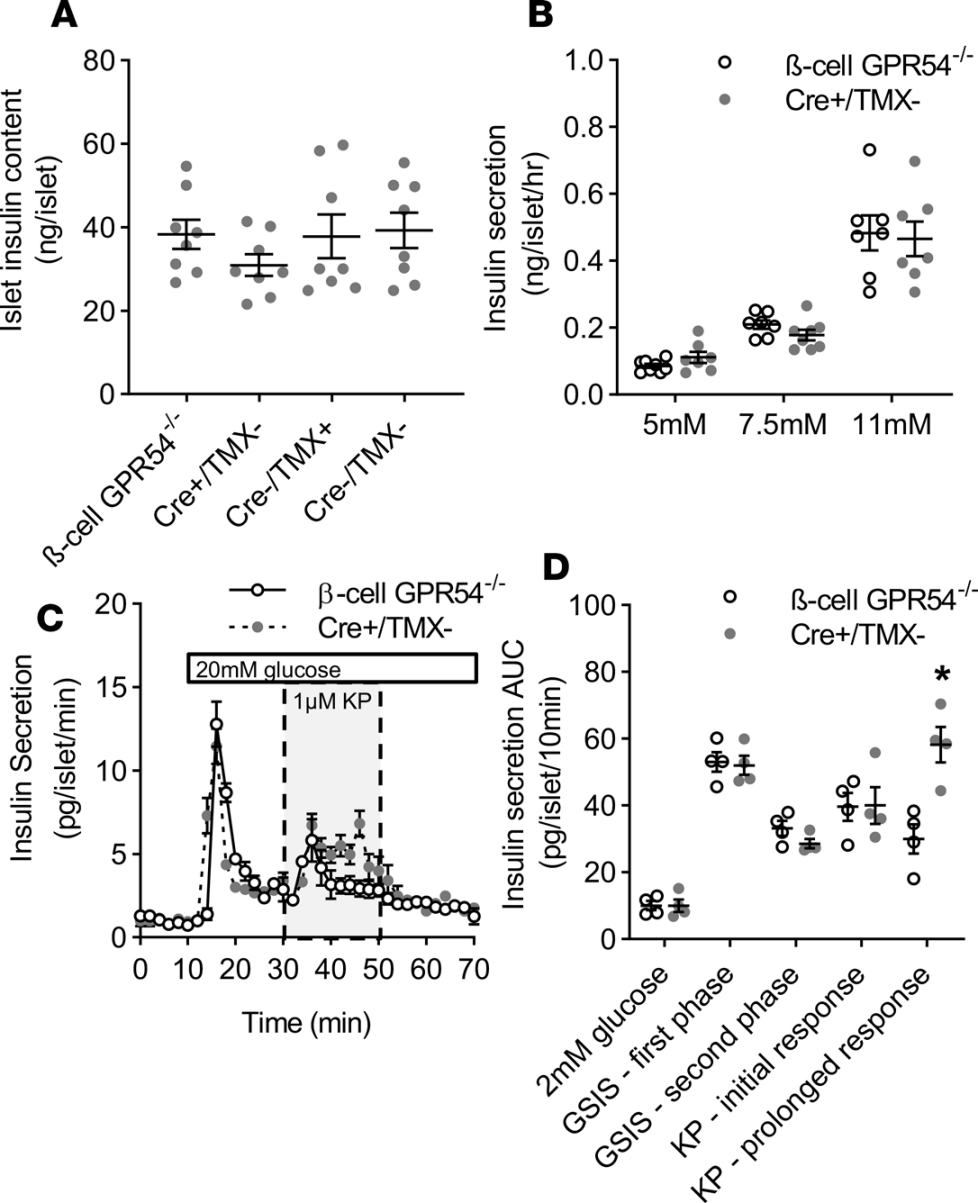
Effects of β cell GPR54 deletion on islet function in vitro. (**A**) There were no significant differences in islet insulin content between β cell GPR54^–/–^ mice and any control groups (Cre^+^/TMX–, Cre^–^/TMX^+^, and Cre^–^/TMX^–^). Mean ± SEM, *n* = 9. (**B**) In static incubation experiments there was no significant difference in the insulin secretory response to physiological glucose concentrations between β cell GPR54^–/–^ and Cre^+^/TMX^–^ islets. (**C**) In perifusion experiments there was no significant difference in basal insulin secretion at 2 mM glucose or first- or second-phase insulin secretion in response to 20 mM glucose in β cell GPR54^–/–^ compared to Cre^+^/TMX^–^ islets. Exposure of Cre^+^/TMX^–^ islets to kisspeptin (1 μM, 30–50 minutes) resulted in a sustained enhancement of second-phase insulin secretion for the duration of kisspeptin administration. However, in β cell GPR54^–/–^ islets this response was transient and not maintained beyond 10 minutes as determined by comparison of individual time points and (**D**) AUC over 10-minute phases of the perifusion (2-tailed Student’s *t* test; KP — prolonged response; *P* = 0.007). Mean ± SEM; *n* = 8 (**A**); *n* = 7–8 (**B**); *n* = 4 (**C** and **D**); data representative of experiments conducted 2–3 times; *P < 0.05.

For studies in β cell GPR54^–/–^ mice, 3 control groups were potentially necessary: Cre^+^ mice not administered tamoxifen (Cre^+^/TMX–), Cre^–^ mice administered tamoxifen (Cre^–^/TMX^+^), and Cre^–^ mice not administered tamoxifen (Cre^–^/TMX^–^). There was no difference in insulin content between β cell GPR54^–/–^ mice and any of these control groups (Cre^+^/TMX^–^, Cre^–^/TMX^+^, and Cre^–^/TMX^–^; Figure 3A). For subsequent in vitro characterization the Cre^+^/TMX^–^ group was used because the presence of the Cre gene was more likely to represent a confounding factor in terms of islet function. In static incubation experiments there was no significant difference between β cell GPR54^–/–^ and Cre^+^/TMX^–^ islets in glucose-stimulated insulin secretion across a physiological range of glucose concentrations from 5 to 11 mM glucose (Figure 3B). Similarly, in perifusion experiments there was no difference in basal insulin secretion at 2 mM glucose or in the first- or second-phase insulin response to a maximally stimulating glucose concentration (20 mM; Figure 3, C and D). In Cre^+^/TMX^–^ islets the addition of 1 μM kisspeptin-10 also resulted in a significant potentiation of glucose-stimulated insulin secretion, which was sustained for the duration of kisspeptin exposure. In contrast, β cell GPR54^–/–^ islets showed an initial stimulatory insulin response to kisspeptin, but this effect was brief; kisspeptin was unable to maintain a prolonged potentiating effect (Figure 3, C and D).

### Effects of deletion of β cell GPR54 on glucose homeostasis in vivo.

**Figure 4:**
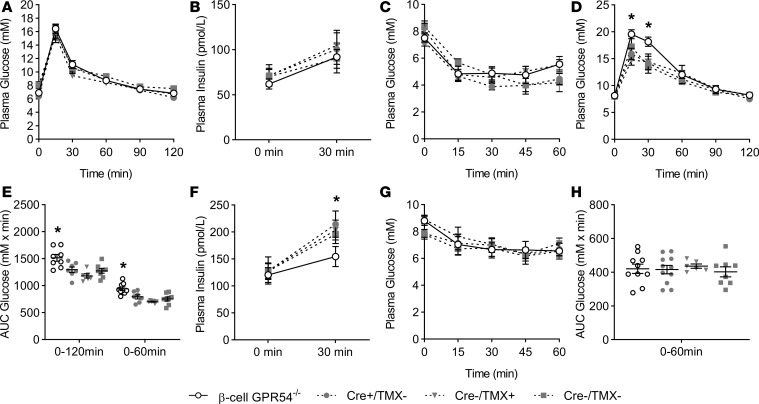
Glucose tolerance in female β cell GPR54^–/–^ mice. (**A**) There was no significant difference in glucose tolerance between nonpregnant adult female β cell GPR54^–/–^ mice and any of the female control groups (Cre^+^/TMX^–^, Cre^–^/TMX^+^, and Cre^–^/TMX^–^). Similarly, the β cell GPR54^–/–^ mice did not have (**B**) significantly altered plasma insulin levels, either fasted or after glucose, or (**C**) any change in insulin resistance when compared to any control group. (**D**) At day 16 of pregnancy β cell GPR54^–/–^ mice had significantly impaired glucose tolerance at 15 and 30 minutes after glucose administration (2-way repeated-measures ANOVA; 15 minutes *P* = 0.028; 30 minutes *P* = 0.014) and (**E**) glucose AUC over the course of the test when compared with all control groups (1-way ANOVA; 0–120 minutes AUC *P* = 0.02; 0–60 minutes AUC *P* = < 0.001). (**F**) Pregnant β cell GPR54^–/–^ mice did not have significantly altered basal fasting plasma insulin levels at day 16 of pregnancy; however, GPR54 knockdown did result in significantly reduced insulin release in response to i.p. glucose administration (2  g/kg) after 30  minutes when compared with all controls (1-way ANOVA, *P* = 0.045). (**G**) Pregnant β cell GPR54^–/–^ mice did not have significantly different insulin sensitivity either at any individual time point or in (**H**) glucose AUC. Mean ± SEM; *n* = 7–9 (**A**–**C**); *n* = 7–12 (**D**–**H**); *P < 0.05.

Having confirmed normal glucose responsiveness, but impaired kisspeptin response, in β cell GPR54^–/–^ islets, the mice were used to assess whole-body glucose homeostasis. GPR54 ablation in β cells had no significant effects in nonpregnant mice, as shown in Figure 4, A–C. Thus, virgin female β cell GPR54^–/–^ mice showed no significant differences in glucose tolerance (Figure 4A), glucose-induced insulin secretion (Figure 4B), or insulin resistance (Figure 4C) when compared to any of the 3 control groups (Cre^+^/TMX^–^, Cre^–^/TMX^+^, and Cre^–^/TMX^–^). In addition, we did not detect any significant differences in glucose tolerance, circulating insulin, or insulin sensitivity (Figure 4, A–C) between any of the control groups, suggesting that neither the presence of the Cre transgene nor the use of TMX as an activator had any significant effect on glucose homeostasis in this experimental model.

However, the β cell GPR54^–/–^ mouse model confirmed the importance of kisspeptin signaling in β cell adaptive responses to pregnancy, as shown in Figure 4, D–H. Thus, by day 16 of pregnancy β cell GPR54^–/–^ mice had significantly impaired glucose tolerance when compared with controls (Figure 4, D and E), and this was associated with reduced insulin secretory responses 30 minutes after a glucose challenge (Figure 4F), although basal plasma insulin levels were unaffected by the β cell GPR54 deletion (Figure 4F). In accordance with the studies using the kisspeptin antagonist in pregnant mice (Figure 2, D and E), pregnant β cell GPR54^–/–^ mice showed no significant differences in insulin resistance (Figure 4, G and H) or in body weight when compared to controls (Supplemental Figure 4A). Thus, deletion of β cell GPR54 induces glucose intolerance in pregnancy by reducing glucose-induced insulin secretion, consistent with placental kisspeptin acting to compensate for pregnancy-induced insulin resistance by enhancing β cell function.

### Effects of deletion of β cell GPR54 on pregnant β cell mass.

**Figure 5:**
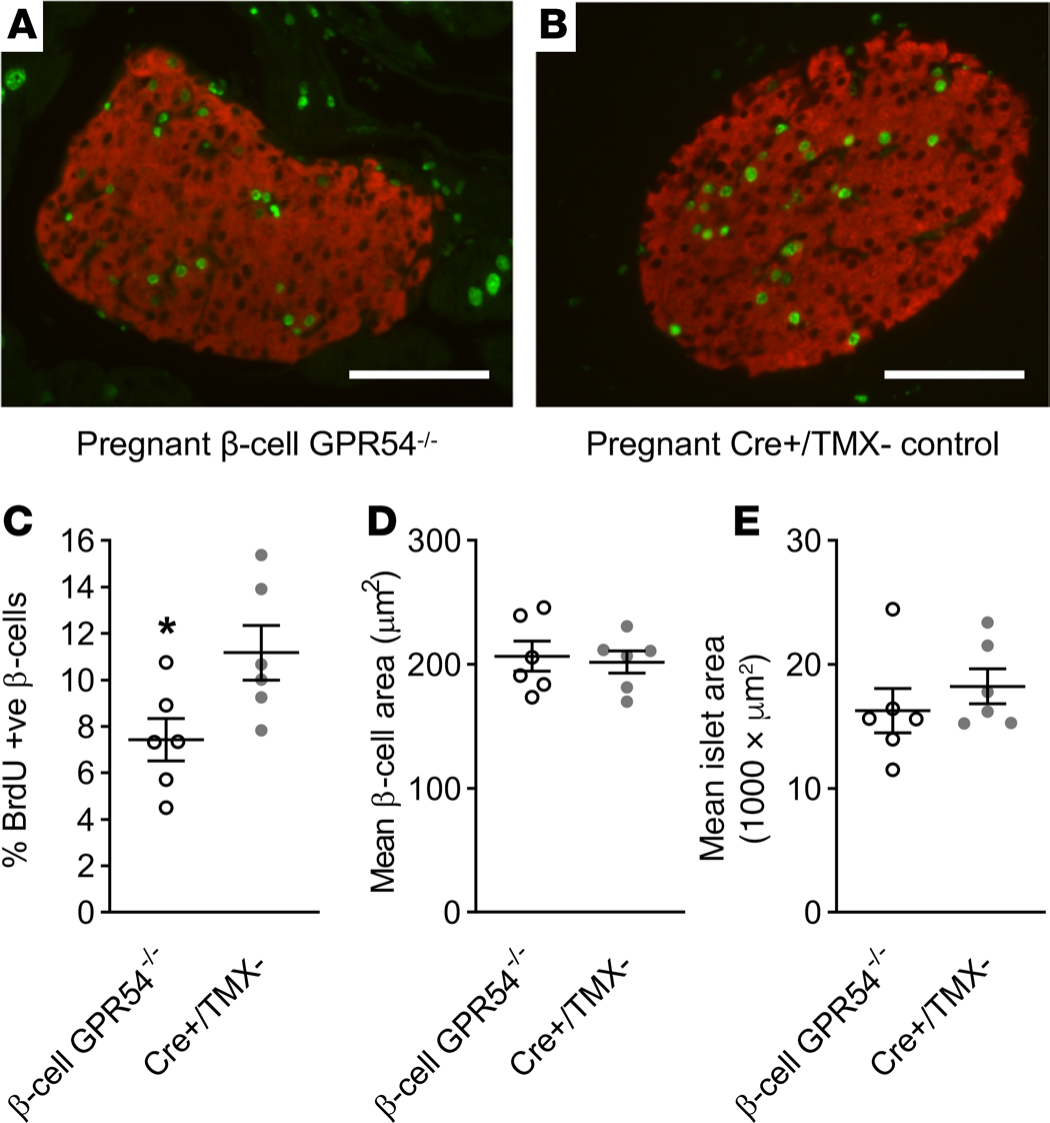
Effects of deletion of β cell GPR54 on pregnant β cell mass. Representative illustrative images of immunostaining for the measurement of β cell proliferation in pregnant (**A**) β cell GPR54^–/–^ and (**B**) Cre^+^/TMX^–^ islets showing merged BrdU staining (shown in green) and insulin staining (shown in red). Scale bars: 100 μm. (**C**) β cell GPR54^–/–^ mice administered BrdU from days 10–18 of pregnancy had significantly reduced levels of BrdU labeling compared with pregnant Cre^+^/TMX^–^ mice (2-tailed Student’s *t* test, *P* = 0.041). At day 18 of pregnancy there was no significant difference in either (**D**) mean overall islet area or (**E**) size of individual β cells between β cell GPR54^–/–^ and Cre^+^/TMX^–^ mice. Mean ± SEM; *n* = 6 (**C**–**E**); *P < 0.05.

Pregnant mice were administered BrdU from days 10–18 of pregnancy to label proliferating cells before pancreas samples being collected at day 18. The percentage of BrdU^+^ β cells was significantly reduced in β cell GPR54^–/–^ mice compared with Cre^+^/TMX^–^ controls, demonstrating an attenuation of the rapid β cell proliferation normally observed during late pregnancy (Figure 5, A–C). Despite the change in β cell proliferation, there was no significant difference in islet cross-sectional area between day 18 pregnant β cell GPR54^–/–^ mice and Cre^+^/TMX^–^ controls (Figure 5D).

Rodent pregnancy is also associated with β cell hypertrophy, so the mean individual β cell area was calculated as total β cell area divided by number of β cell nuclei. This may slightly overestimate individual β cell area because not all β cell nuclei may be visualized in a given section, but it is still a useful measure for comparing changes in individual β cell area between treatment groups. No significant difference was observed in the mean area of individual β cells, suggesting that deletion of β cell GPR54 has no effect on pregnancy-induced hypertrophy (Figure 5E).

### Circulating kisspeptin and glucose tolerance in pregnant women.

**Figure 6:**
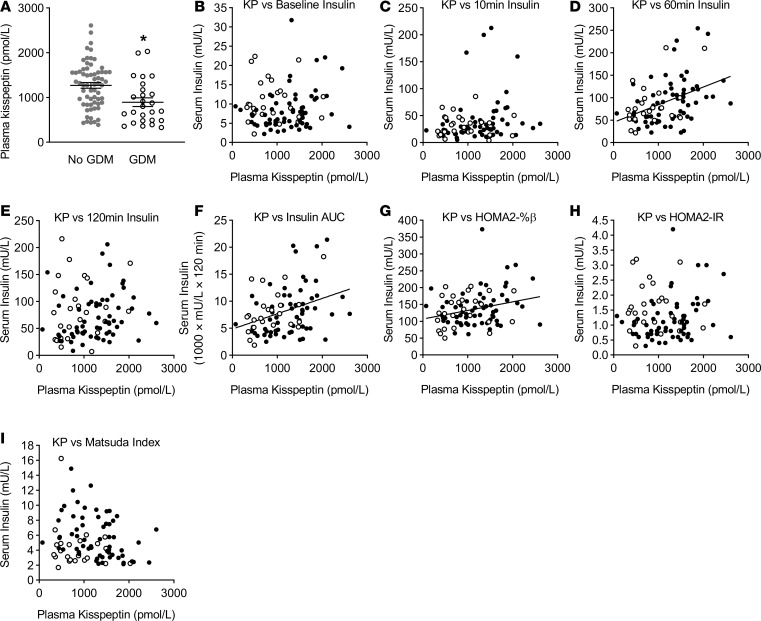
Relationships between kisspeptin and insulin response to oral glucose and presence of GDM in pregnant women. (**A**) In pregnant women undergoing a routine 75-g oral glucose tolerance test (OGTT), women with gestational diabetes mellitus (GDM, International Association of Diabetes and Pregnancy Study Groups [IADPSG] 2010 criteria, *n* = 26) had significantly lower kisspeptin than women without GDM (*n* = 62; *P = 0.0022; 2-tailed Student’s *t* test). (**B**) Analyzing all pregnant women (*n* = 91) there was no significant correlation between kisspeptin levels and fasting serum insulin, but there was a significant positive correlation between kisspeptin and serum insulin at 60 minutes (**D**; *r*^2^ = 0.1757; *P* < 0.0001) and between kisspeptin and AUC serum insulin over the OGTT (**F**; *r*^2^ = 0.1279; *P* = 0.0013). There was no significant correlation between kisspeptin and serum insulin at 10 minutes (**C**) or 120 minutes (**E**). There was a significant positive correlation between kisspeptin and HOMA2-%β (**G**; *r*^2^ = 0.0656, *P* = 0.0411), but no significant correlations between kisspeptin and HOMA2-IR (**H**) or the Matsuda index (**I**). Women diagnosed with GDM are represented by white markers; women without GDM are represented by black markers. Pearson product-moment correlation coefficient was used for analyzing correlation data, and presented correlation data are based on all women. Correlation coefficient lines denote a significant correlation between variables.

Our studies in a cohort of pregnant women further support a role for circulating placental kisspeptin in β cell adaptive responses to pregnancy, as shown in Figure 6. Under basal (fasting) conditions there was a significant, albeit weak, positive correlation between kisspeptin and β cell secretory function, as assessed by Homeostatic Model Assessment 2–% β cell function (HOMA2-%β) (Figure 6G), although there was no significant correlation with fasting insulin (Figure 6B). There was a highly significant medium-strength positive correlation between kisspeptin and oral glucose–stimulated insulin levels, as assessed by insulin AUC (Figure 6F). There was a highly significant medium-strength positive correlation between kisspeptin and serum insulin at 60 minutes after oral glucose load (Figure 6D), with a trend toward significance at 10 minutes (Figure 6C) and no significant correlation at 120 minutes (Figure 6E). There was a slight negative correlation between kisspeptin and fasting plasma glucose (*r*^2^ = –0.064; *P* = 0.016), with no significant correlations at 10 minutes, 60 minutes, or 120 minutes (Supplemental Figure 5), suggesting the positive correlations between kisspeptin and insulin are not secondary to higher circulating glucose levels. There were no significant correlations between kisspeptin and Homeostatic Model Assessment 2–insulin resistance (HOMA2-IR) (Figure 6H) or the Matsuda index (Figure 6I), suggesting the positive correlations between kisspeptin and insulin are not secondary to altered insulin resistance.

In this cohort, 28.6% of women had GDM. There were no significant differences in age, ethnicity, multiple pregnancy, gestation, number of previous pregnancies, BMI, or blood pressure between women with and without GDM (Supplemental Table 1). However, women with GDM had significantly lower plasma kisspeptin levels than women without GDM (Figure 6A).

The reported correlations are for all pregnant women, both with and without GDM. Correlation coefficients were also calculated for women with and without GDM separately, and *r*^2^ values for the independent groups were similar to those for the combined data. The correlation observed between kisspeptin and HOMA2-%β was no longer significant when comparing women with GDM alone; however, this is most likely due to the smaller *n* value in the GDM group and the initial weak correlation. All other correlations that were significant in the combined data were also significant for women with and without GDM independently.

In summary, in pregnant women higher plasma kisspeptin levels are associated with enhanced insulin secretion, particularly after oral glucose load, and lower levels of plasma kisspeptin are associated with diagnosis of GDM.

## Discussion

Pregnancy represents a unique physiological state in which the islet β cells are exposed to very high levels of circulating kisspeptin for a prolonged period, and we have here demonstrated that placentally derived kisspeptin is involved in β cell adaptations to pregnancy. Chronic elevations in circulating kisspeptin in virgin mice improved glucose tolerance as a consequence of enhanced glucose-induced insulin secretion, consistent with previous in vitro studies of a direct stimulatory effect of β cell GPR54 activation on insulin secretion (7). The significant effects of exogenous kisspeptin on glucose tolerance in nonpregnant mice were only apparent between 15 and 30 minutes after glucose administration, most likely because the normal control of blood glucose leaves little room for improvement, but the chronic kisspeptin treatment had marked effects to enhance glucose-induced insulin secretion in vivo. During murine pregnancy, pharmacological blockade of GPR54 resulted in impaired glucose tolerance as a consequence of reduced glucose-induced insulin secretion. In normal mouse pregnancy, glucose tolerance is progressively impaired from mid-pregnancy (days 10–12) to late pregnancy (day 16–18) and associated with increased peripheral insulin resistance. Thus, pharmacological blockade of GPR54 induced a mild impairment of glucose tolerance at mid-pregnancy, which progressed to a more pronounced phenotype by late pregnancy, suggesting a progressive role for endogenous kisspeptin in mediating β cell adaptations to pregnancy-induced insulin resistance. This is consistent with the development of the mouse placenta, which becomes fully functional only around days 7–9 (27), suggesting that levels of circulating kisspeptin are unlikely to be elevated before this stage, with a subsequent progressive increase in circulating kisspeptin for the remainder of the pregnancy, as has been reported during human pregnancy (17). The precise changes in circulating levels of kisspeptin in rodent pregnancy have not been clearly established because of continuing difficulties in developing accurate and specific rodent assays. However, rodent placenta expresses increasing levels of kisspeptin mRNA through pregnancy (18–20), consistent with observations in humans, suggesting placental release of kisspeptin into the maternal circulation.

The pharmacological data were supported by our observations using a β cell–specific GPR54-knockout mouse model. Hypothalamic GPR54 plays a critical role in maintaining reproductive function, so the B6.Cg-Tg(Ins1-cre/ERT)1Lphi/J (MIP-CreERT) line was selected to avoid the off-target hypothalamic Cre expression that is a characteristic of the PDX1-Cre and RIP-Cre lines (28, 29). Although β cell–specific GPR54-knockout mice have been previously reported (13), they have not previously been studied during pregnancy. Validation of the β cell GPR54^–/–^ mice demonstrated a tissue-specific 70% reduction in islet GPR54 mRNA expression, with no detectable leakage in the hypothalamus (see Supplemental Figure 3). This reduction in GPR54 mRNA expression is consistent with other inducible knockout models, where 100% gene knockout is not typically observed. Also, there is some controversy regarding whether GPR54 is also expressed in islet α cells, with some previous studies suggesting it is expressed (4) and others finding it absent (13). If GPR54 is present in islet endocrine cells other than β cells, then this would not be deleted in the β cell GPR54^–/–^ mice, resulting in an underestimation of GPR54 knockdown based on whole islet expression. In vitro studies in isolated islets confirmed that GPR54 knockdown had no effect on insulin content or on glucose-induced insulin secretion. Kisspeptin treatment of β cell GPR54^–/–^ islets did still induce a brief, transient potentiation of insulin secretion, which may be consistent with the presence of residual GPR54 expression in the islets. However, kisspeptin did not maintain elevated insulin release in β cell GPR54^–/–^ islets, suggesting that the effects are significantly attenuated in β cell GPR54^–/–^ islets.

Based on the GPR54 blockade studies, β cell GPR54^–/–^ mice were assessed during late pregnancy (days 16–18) to maximize any metabolic phenotype. As expected, pregnant β cell GPR54^–/–^ mice had significantly impaired glucose tolerance during late pregnancy as a consequence of reduced glucose-induced insulin secretion, in accordance with the pharmacological studies and supporting a β cell–specific action of placental kisspeptin to regulate glucose tolerance. However, no differences in placental weight, pup weight, or pup length were observed between β cell GPR54^–/–^ mice and controls (Supplemental Figure 4, B–D). The absence of macrosomia suggests that despite the significant glucose intolerance, loss of β cell GPR54 signaling is not sufficient to induce GDM.

These studies used several controls to avoid any potentially confounding factors associated with the inducible transgene. Thus, the MIP-CreERT mouse has been reported to exhibit inappropriate human growth hormone expression in β cells, leading to increased islet serotonin levels (30–32), an effect that has so far been demonstrated only in males and that is relatively small compared with the normal increases in islet serotonin seen during pregnancy (3, 33). Nonetheless, we used Cre^+^ control mice to demonstrate that the transgene itself had no effect on glucose tolerance in pregnant or nonpregnant female mice. Tamoxifen is a selective estrogen receptor modulator that can disrupt female reproductive cycles (34) and affect glucose tolerance through effects on lipid metabolism (30, 34, 35). To minimize these confounding effects, mice were left for 6 weeks following TMX administration, by which time animals had regained fertility and no TMX-dependent effects on glucose tolerance were detectable.

In addition to enhanced secretory responsiveness to glucose, β cells also adapt to pregnancy by increasing their functional β cell mass, through a combination of an increased rate of proliferation and β cell hypertrophy in rodents. This β cell expansion occurs primarily after placentation in rodents over a relatively short and well-defined time span and is reversible postpartum, consistent with placental signals playing a major role in informing the β cells of the gestational stage via circulating mediators. It is well established that the lactogenic hormones, prolactin and placental lactogen, play a key role in metabolic adaptations during pregnancy, but lactogenic hormones are unlikely to account for all the β cell adaptive responses; other factors have been implicated in the successful islet adaptation to pregnancy (36). Our observations of reduced β cell proliferation in pregnant β cell GPR54^–/–^ mice suggest that placental kisspeptin does play a physiological role in regulating β cell mass to compensate for the pregnancy-induced insulin resistance. However, the reduced proliferative rate in pregnant β cell GPR54^–/–^ islets is not associated with a corresponding reduction in β cell hypertrophy and overall is not sufficient to translate into a significant overall reduction in β cell mass. It might be expected that the reduced rate of proliferation would result in a corresponding reduction in mean islet area. Although there is a trend toward reduced islet area in the pregnant β cell GPR54^–/–^ mice, it is likely that the relatively small change in proliferation rate combined with the natural variability in islet area means that any effect on islet area is not sufficient to achieve significance. It is worth noting that, though significant, the effects of β cell GPR54 knockdown on proliferation are not as dramatic as the effect observed in response to β cell–specific knockdown of the prolactin receptor (37). Thus, although placental kisspeptin may play a contributory role in the upregulation of β cell proliferation during pregnancy, it does not appear to be a major driver of this response to the extent of the lactogenic hormones.

Our mouse data suggest that kisspeptin signaling via GPR54 facilitates metabolic control by enhancing the insulin secretory responses of the β cells and to some extent by increasing the number of β cells. Less information is available about the regulation of the β cell adaptation to human pregnancy, particularly with regard to β cell mass. Postmortem studies indicate that β cell mass certainly increases in human pregnancy, though the underlying mechanisms are unclear and may differ from rodent pregnancy (38). Regardless, the core characteristics involved in the β cell adaptation in rodents appear to be conserved to some extent in human pregnancy.

There is great interest in determining whether signals identified in rodent pregnancy are also involved in regulating human β cells. Our previous studies suggested that the effects of kisspeptin in pregnancy may translate to humans, given that kisspeptin is able to potentiate glucose-stimulated insulin secretion from human islets in vitro (6). Our studies in pregnant women undergoing OGTTs as part of their routine clinical care suggest that placental kisspeptin may have a similar function in humans to that revealed in our mouse models. In accordance with our animal data, glucose-induced insulin secretion was positively correlated with plasma kisspeptin. This is consistent with placentally derived kisspeptin exerting an “incretin-like” (39) action in pregnancy to facilitate greater β cell insulin secretion in response to glucose ingestion to compensate for pregnancy-associated insulin resistance. We also observed a weak positive correlation between kisspeptin and basal β cell secretory function as assessed by HOMA2-%β in pregnant women, though there was no significant correlation between kisspeptin and fasting insulin levels in either pregnant women or in animal studies. In addition, we demonstrated that women with GDM had significantly lower plasma kisspeptin levels than women without GDM, which supports a previous report of lower mean kisspeptin in GDM (40). Low placental production of kisspeptin has been suggested as a risk factor for preeclampsia and early pregnancy bleeding (41, 42), and it is of interest that GDM is a risk factor for preeclampsia (43). These data are constrained by the obvious ethical limits of intervening during human pregnancy, and there should be some caution when drawing conclusions from correlative results. However, it is noteworthy that all the significant correlations observed in these pregnant women are between kisspeptin levels and markers of β cell function (e.g., robust insulin response to glucose, HOMA2-%β), with no correlations observed between kisspeptin and other markers (e.g., HOMA2-IR). Considered with our animal data, this suggests reduced placental kisspeptin production, with consequent impaired kisspeptin-dependent β cell compensation, may be a factor in the development of GDM in humans.

It is less clear what, if any, role kisspeptin plays in metabolic control outside of pregnancy because we detected no phenotype in nonpregnant β cell GPR54^–/–^ mice. Previous studies have reported both potential beneficial and inhibitory effects of kisspeptin on glucose tolerance in nonpregnant, usually male, mice (13, 14, 44), but these effects may not be β cell specific and are most often observed in metabolically stressed animals. Furthermore, a recent human study reports that elevated plasma kisspeptin correlates with impaired glucose tolerance in both male and female, nonpregnant, nondiabetic individuals (45), though it is well established that plasma kisspeptin levels are several thousand–fold lower outside of pregnancy (17). Although we cannot rule out that kisspeptin may play a role in regulating β cell function outside of pregnancy, the impaired glucose tolerance seen in these β cell GPR54^–/–^ mice appears to be a pregnancy-specific phenomenon. It has been suggested that the different-length kisspeptin peptides may have differing effects, and it is worth noting that the placenta releases all forms of kisspeptin into the circulation. There is a precedent for the differing forms of kisspeptin having specific effects because kisspeptin-10, but not longer forms of kisspeptin, plays a role in trophoblast invasion (46). Several studies have put forward the hypothesis that higher concentrations of kisspeptin may potentiate glucose-induced insulin release while lower concentrations may be inhibitory (47). As such, our findings in pregnant women and the β cell GPR54^–/–^ mouse model do not necessarily contradict previous studies demonstrating inhibitory effects of kisspeptin on insulin secretion but support the possibility that kisspeptin may have different effects under different conditions, though the precise mechanisms involved remain to be established.

Together, our data are consistent with placentally derived kisspeptin in the maternal circulation exerting an effect on maternal β cells during pregnancy to facilitate enhanced glucose-induced insulin secretion and increase β cell mass to compensate for maternal insulin resistance, thus avoiding overt hyperglycemia. The expression of GPR54 by β cells allows them to detect elevations in kisspeptin during pregnancy, informing them about the state of pregnancy and enabling them to respond to the peculiar metabolic demands of pregnancy. We have recently reported that mouse placenta expresses about 80 ligands for β cell GPCRs (48), suggesting that kisspeptin may be one of many novel placental signals regulating β cell function during pregnancy.

In conclusion, we have demonstrated an important role for placental kisspeptin in amplifying glucose-induced insulin secretion across species, consistent with a physiological role for kisspeptin in the β cell adaptation to pregnancy. Our data in pregnant women, considered with our animal data, suggest reduced placental kisspeptin production, with consequent impaired kisspeptin-dependent β cell compensation, may be a factor in the development of GDM in humans. Kisspeptin may offer a promising biomarker for developing GDM, or a tractable target for therapeutic intervention, or both.

## Methods

### Animals.

Female ICR mice (Envigo, Bicester, United Kingdom) at 8 weeks of age were used for in vivo pharmacological studies. The β cell GPR54^–/–^ mouse model was generated on a C57BL/6 background using Cre-lox recombination. GPR54-*loxP* mice (49) (obtained from M. Tena-Sempere, University of Cordoba, Spain) were crossbred with MIP-Cre/ERT mice (ref. 50; Jackson Labs). TMX was administered to induce GPR54 knockdown in the relevant mice at 8 weeks of age through daily i.p. injection (100 μL of 20 mg/mL TMX in peanut oil, MilliporeSigma) for 4 consecutive days. Mice were then left for 6 weeks to allow TMX washout. Further details on the validation of the β cell GPR54^–/–^ mice are available within Supplemental Figure 2.

All animals were housed under controlled conditions (14-hour light/10-hour dark, lights on at 0700 hours, temperature at 22 ± 2°C) and provided food and water ad libitum.

### In vivo studies.

For pregnant mice, females were housed with a male and checked daily for the presence of a vaginal plug. The day a vaginal plug was observed was designated day 0 of pregnancy. Age-matched female mice were used for studies in nonpregnant animals.

For pharmacological studies, animals were implanted with a subcutaneous osmotic minipump (Alzet) under isoflurane anaesthesia. Minipumps contained either 1 mM of kisspeptin-10 (Alta Biosciences) or 2 mM of the GPR54 antagonist kisspeptin-234 (51) (MilliporeSigma). In pregnant animals minipumps were implanted at either day 2 or day 8 of pregnancy. Osmotic minipumps released either 0.25 nmol/h kisspeptin-10 or 0.4 nmol/h kisspeptin-234 at an infusion rate of 0.4 nmol/h.

For glucose tolerance tests mice were fasted for 6 hours and administered glucose (2 g/kg) via i.p. injection. Blood samples (~1 μL) were taken by tail prick for the measurement of blood glucose levels. Larger blood samples were taken from the tail vein for the measurement of plasma insulin levels. Plasma insulin levels were subsequently measured using a mouse ultrasensitive insulin ELISA kit (Mercodia).

For insulin tolerance tests mice were also fasted for 6 hours and were administered insulin (0.75 IU/kg, MilliporeSigma) via i.p. injection. Small blood samples were taken by tail prick for the measurement of blood glucose levels.

In β cell GPR54^–/–^ mice, from day 10 of pregnancy onward, BrdU (1 mg/mL) was administered in the drinking water to label proliferating cells. Following the insulin tolerance test animals were culled and the pancreas collected for histology.

### β cell GPR54^–/–^ in vitro studies.

Islets of Langerhans were isolated from female β cell GPR54^–/–^ and control mice by collagenase digestion of the exocrine pancreas. Islets were incubated at 37°C in RPMI (supplemented with 10% fetal calf serum, 2 mM glutamine, and 100 units/mL penicillin/0.1 mg/mL streptomycin) for at least 24 hours before use.

Insulin secretion from mouse islets was assessed either using a temperature-controlled perifusion apparatus or in static incubations of islets. For perifusion experiments groups of 40 islets were perifused with a bicarbonate-buffered physiological salt solution (0.5 mL/min, 37°C) containing 2 mM glucose, 2 mM CaCl_2_, and 0.5 mg/mL BSA and supplemented with treatments of interest. Perifusate was collected at 2-minute intervals for insulin content. For static incubations islets were preincubated in 2 mM glucose RPMI. Groups of 10 size-matched islets were incubated at 37°C for 1 hour in physiological salt solution as described above, supplemented with agents of interest. After 1 hour samples were taken and assayed for insulin content. For islet insulin content, groups of 10 islets were transferred into acid-ethanol and sonicated. Insulin levels were determined using an in-house radioimmunoassay.

### β cell mass analysis.

Pancreas samples were fixed in 4% paraformaldehyde (MilliporeSigma) and embedded in paraffin wax. Sections (5 μm) were rehydrated followed by antigen retrieval with 2N hydrochloric acid for 20 minutes at 37°C and subsequent 0.05% trypsin solution (MilliporeSigma) for 15 minutes at 37°C. Sections were incubated in blocking buffer (0.25% BSA, 0.25% Triton X-100 in PBS) before incubation with both a mouse monoclonal anti-BrdU antibody (1:100, MilliporeSigma, B8434) and guinea pig polyclonal anti-insulin antibody (1:200, Dako, A0564) for 2 hours at 37°C.

Alexa Fluor 488–conjugated donkey antimouse (1:100, Jackson ImmunoResearch) and Alexa Fluor 594–conjugated donkey anti–guinea pig (1:100, Jackson ImmunoResearch) were used to visualize staining. A Nikon Eclipse TE2000-U microscope was used for acquiring images at original magnification ×200 using a Nikon DS-Qi1MC camera.

Sections were analyzed from across the pancreas for each animal, and all islets on each section were analyzed. ImageJ image analysis software (NIH) was used to count the number of BrdU^+^ β cells, total number of β cells, and cross-sectional area for each islet.

### Plasma kisspeptin and glucose tolerance in pregnant women.

Pregnant women between 26 and 34 weeks’ gestation referred for an OGTT at King’s College Hospital as part of routine care were invited to participate. Women were referred for an OGTT if they had GDM in a previous pregnancy, BMI at least 40 kg/m^2^, or random plasma glucose at least 6.7 mmol/L. Exclusion criteria were being unable to give informed consent and known major medical problems. One hundred women were recruited and data are presented for 91 women for whom blood samples were obtained. Further participant details are available within Supplemental Table 1.

Participants underwent a standard 2-hour 75-g OGTT with additional blood sampling. After overnight fasting (>9 hours), an intravenous cannula was inserted in an arm vein for blood sampling, the participant drank 75 g glucose in 300 mL, and rested for 2 hours. Venous blood samples were taken before glucose consumption for the measurement of plasma kisspeptin, plasma glucose, and serum insulin. Further blood samples were taken at 10 minutes, 60 minutes, and 120 minutes for the measurement of plasma glucose and serum insulin. For this research GDM was diagnosed according to the IADPSG criteria (52): 1 or more of venous plasma glucose fasting ≥ 5.1 mmol/L, 60 minutes ≥ 10.0 mmol/L, or 120 minutes ≥ 8.5 mmol/L. Weight, height, and blood pressure were measured.

### Preparation and assay of human samples.

For plasma kisspeptin, 5-mL venous blood samples were collected into tubes containing EDTA (BD Vacutainer Blood Collection Tubes) (53). Samples were centrifuged at 855 *g* for 10 minutes at 4˚C. Plasma was collected and stored at –80˚C until assay. Plasma kisspeptin was measured using a commercially available ELISA kit (Phoenix Pharmaceuticals Inc.). Peptide extraction was performed on all kisspeptin samples based on the manufacturer’s protocol (Phoenix Pharmaceuticals Inc.) and absorbances measured using a Chameleon plate reader.

For serum insulin, 5-mL venous blood samples were collected into SST tubes (BD Vacutainer Blood Collection Tubes) and allowed to stand for a minimum of 20 minutes at room temperature before centrifugation at 855 *g* for 10 minutes at 4˚C. Serum was collected and stored at –80˚C until assay. Serum insulin levels were measured using a commercially available ELISA kit (Mercodia). The ELISA was performed according to the manufacturer’s instructions and absorbances measured using a Chameleon plate reader.

For plasma glucose measurement, 2-mL venous blood samples were collected into tubes containing Fluoride EDTA (BD Vacutainer Blood Collection Tubes). Samples were centrifuged at 855 *g* for 10 minutes at 4˚C. Plasma glucose was measured immediately using a YSI 2300 Stat Analyser (YSI Life Sciences).

HOMA2-%β and HOMA2-IR were calculated using the HOMA2 calculator (version 2.2.3, specific insulin) downloaded from http://www.dtu.ox.ac.uk/homacalculator/ on August 4, 2018 (54). Matsuda index was calculated using the online calculator available at http://www.mmatsuda.diabetes-smc.jp/MIndexsi.html on August 4, 2018, using time 0, 60, and 120 minutes data (55).

### Statistics.

All data are expressed as mean ± SEM. Each in vitro and in vivo study was repeated at least twice with consistent results. For comparison between 2 groups unpaired 2-tailed Student’s *t* test was used. For comparisons between 3 or more groups, a 1-way ANOVA was used followed by Tukey’s post hoc test. For analysis of individual intraperitoneal glucose tolerance test and intraperitoneal insulin tolerance test data points, 2-way repeated measures ANOVA was used, followed by Tukey’s where appropriate. For analysis of correlations the Pearson product-moment correlation coefficient was used. Differences with *P* less than 0.05 were considered significant.

### Study approval.

All animal procedures were approved by the local King’s College London Animal Welfare and Ethical Review Board and were undertaken in accordance with United Kingdom Home Office Regulations.

The research in humans was conducted in accordance with the Declaration of Helsinki (2013) and was approved by the London-Westminster Research Ethics Committee (13/LO/0539). Written informed consent was obtained from all participants.

## Author contributions

JEB was involved in designing the research studies, conducting animal experiments, and writing the manuscript. TGH conducted the majority of the β cell GPR54^–/–^ experiments, assisted with assaying human samples, and analyzed data. KFH was involved in designing and conducting the translational human studies and in writing the manuscript. LIFS and SJSS assisted with β cell GPR54^–/–^ experiments. SAA assisted with designing and conducting the translational human studies. PMJ was involved in designing the study and writing the manuscript.

## Supplementary Material

Supplemental data

## Version 1 10/17/2019

Electronic publication
