# Supplementary material for: A role for placental kisspeptin in β cell adaptation to pregnancy
Source: JCI Insight. 2019 Oct 17;4(20):e124540. doi: 10.1172/jci.insight.124540 (PMC6824306; doi:10.1172/jci.insight.124540)
Supplement: Supplemental data [file jciinsight-4-124540-s086.pdf]

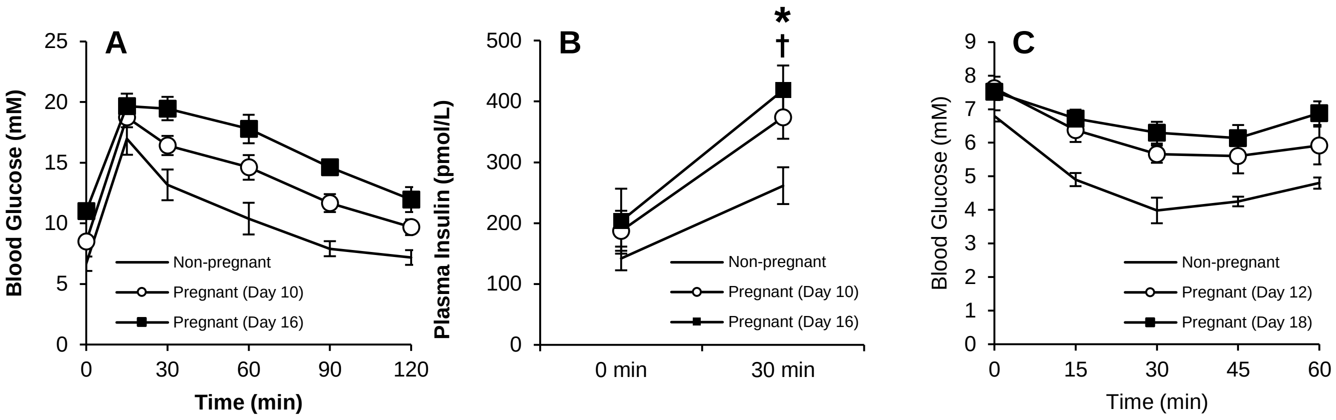

Figure S1: Effects of mid-late pregnancy on glucose homeostasis in mice.

(A) At both mid and late pregnancy (day 10 and day 16 respectively) control mice fasted for 6h had significantly impaired glucose tolerance following i.p. glucose administration (2g/kg) when compared with non-pregnant controls. **(B)** Pregnant control mice also had significantly greater insulin release in response to i.p. glucose administration (2g/kg) after 30min when compared with non-pregnant controls. **(C)** At both mid and late pregnancy (day 12 and day 18 respectively), pregnant control mice fasted for 6h had significantly attenuated reduction in plasma glucose response to i.p. insulin administration (0.75IU/kg) when compared with non-pregnant controls.

Details of β-cell GPR54$^{-/-}$ generation

Experimental β-cell GPR54$^{-/-}$ mice were generated from homozygous GPR54-LoxP males and homozygous GPR54-LoxP / heterozygous MIP-CreERT females. The GPR54-LoxP mice were developed and kindly supplied by Prof. Tena-Sempere at the University of Cordoba and have previously been described in detail (24). The MIP-CreERT mice (Tg (Ins1-Cre/ERT)^{1Lphi}) were developed by Prof Philipson at the University of Chicago and supplied by Jackson Labs. The MIP-CreERT mice have also previously been described in detail (25).

Following two generations of breeding, all experimental mice expressed homozygous GPR54 LoxP and half of experimental mice were heterozygous for the Cre transgene attached to the mouse insulin promoter (MIP). The remaining half of the experimental mice did not carry a copy of the Cre transgene. All experimental mice were allowed to mature beyond puberty and at 8 weeks of age the appropriate mice were administered with tamoxifen to activate the Cre transgene and induce β-cell specific GPR54 knockout, or for control purposes. Tamoxifen was administered by daily intraperitoneal (i.p.) injection (100μl of 20mg/ml tamoxifen in peanut oil, Sigma-Aldrich) for 4 consecutive days. Following tamoxifen administration, mice were then left for a further 6 weeks to allow tamoxifen washout. After 6 weeks, β-cell GPR54-/- mice had normal female reproductive cycles as assessed by vaginal smears and at least 70% β-cell GRP54 knockdown, as assessed by islet mRNA expression.

A

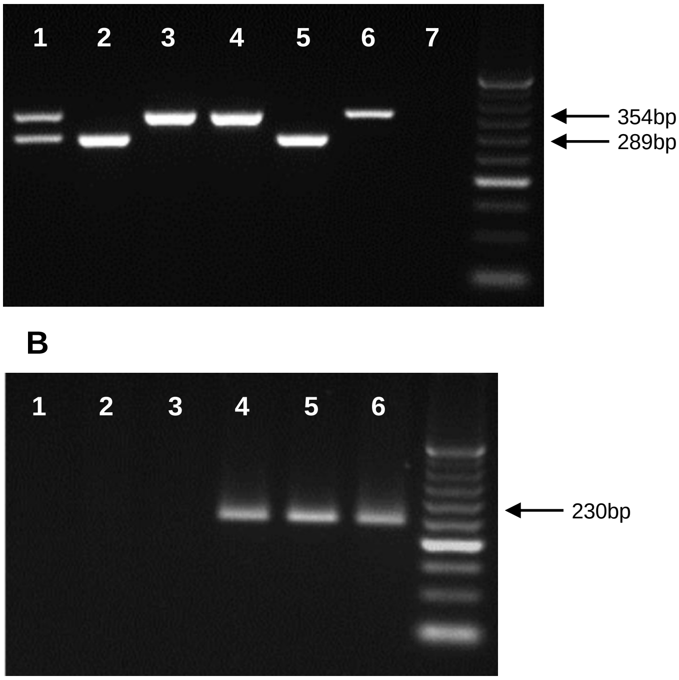

Figure S2: Generation and genotyping of β-cell GPR54$^{-/-}$ mice.

Experimental β-cell GPR54$^{-/-}$ mice were generated from homozygous GPR54-LoxP males and homozygous GPR54-LoxP / heterozygous MIP-CreERT females. The GPR54-LoxP mice were developed and kindly supplied by Prof. Tena-Sempere at the University of Cordoba and have previously been described in detail (24). The MIP-CreERT mice (Tg (Ins1-Cre/ERT)^{1Lphi}) were developed by Prof Philipson at the University of Chicago and supplied by Jackson Labs. The MIP-CreERT mice have also previously been described in detail (25).

For genotyping mouse ear punches were lysed in alkaline lysis buffer (25 mM NaOH/0.2mM EDTA) at 95 ºC, and mixed with 1M Tris (pH 8.0) to isolate the genomic DNA. PCR analysis on isolated genomic DNA was then used to screen both LoxP and Cre genotypes (Figure S1A-B). The primers used for genotyping the LoxP allele were: 5’-AGCGCAAGGCTCTGAAGCGGC-3’ and 5’-AATGTCGCCTCGGTGGCCAT-3’. The primers used to detect the presence of the Cre transgene were: 5’-TGGACTATAAAGCTGGTGGGC-3’ and 5’-TGCGAACCTCATCACTCGT -3’. Amplification was carried out using the following protocol: denaturing for 5 min at 95˚C followed by 30 cycles consisting of denaturing at 95˚C for 30 sec, annealing at 60˚C for 30 sec and extension at 72˚C for 1 min, using a T1 thermal cycler (Bio-Rad laboratories, Hercules, CA). Samples were subsequently analyzed on 2% agarose gels.

GPR54-LoxP primers produce products of different length depending on the presence of the LoxP sequence within the GPR54 gene. In wild-type mice a single product of 289bp is produced. In homozygous GPR54-LoxP mice the presence of the LoxP sequence means that a 354bp product is formed. In heterozygous GPR54-LoxP mice both 289bp and 354bp bands are produced (Fig S1A). For genotyping the Cre transgene, in wild-type mice no PCR product is generated, whilst in Cre+ve mice a single 230bp product is generated. Mice were produced from breeding heterozygous Cre+ve mice with Cre-ve mice, thus a homozygous Cre+ve mouse was not possible. All Cre+ve mice were heterozygous (Fig S1B).

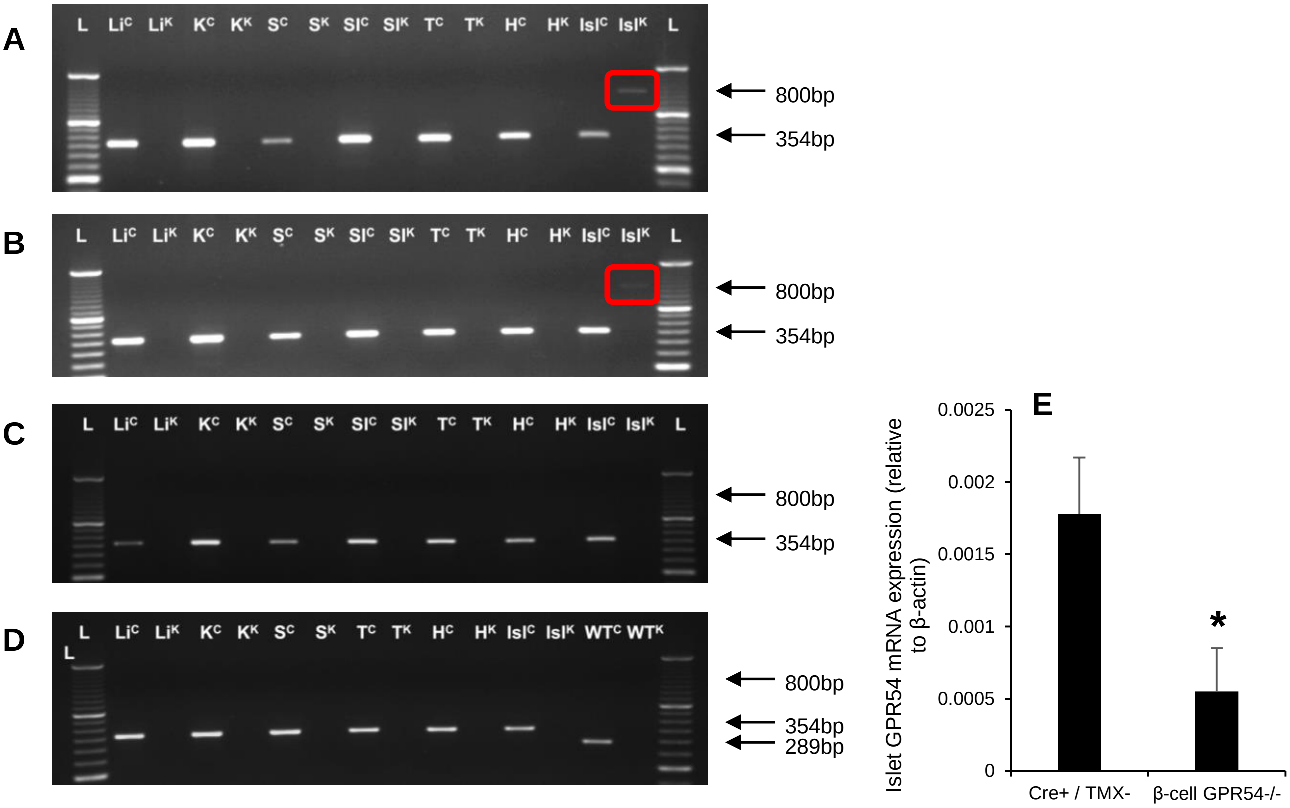

Figure S3: Validation of β-cell GPR54-/- mice.

For validation of successful tissue specific GPR54 knockdown, islet genomic DNA and mRNA was measured. For checking tissue specificity genomic DNA was isolated from liver (Li), kidney (K), spleen (S), small intestine (SI), testes (T), hypothalamus (H) and isolated islets (Isl). Genomic DNA samples were taken from all experimental groups. All genomic DNA samples were processed by PCR utilising sequence-specific primers for both the intact (Control, 'superscript C') and mutated (Knockout, 'superscript K') GPR54 allele. The intact GPR54 gene produces a 354bp amplicon, whilst the primers for the excised GPR54 produce a 800bp product.

(A) In β-cell GPR54-/- mice the intact GPR54 allele is detected in the genomic DNA of all tissues analysed. The 800bp PCR product indicating an excised GPR54 allele is present only in the islet DNA sample (IslK), demonstrating that the knockdown is tissue specific. The islet sample contains both intact and excised GPR54, indicating that there is not complete knockdown of GPR54. (B) In Cre+/TMX- mice there is also a very faint 800bp band in the islet DNA sample (IslK) indicating some excised GPR54 in the absence of TMX. This is expected and previous studies in inducible Cre lines have shown very low levels of Cre activity in the absence of TMX. The (C) Cre-/TMX+ and (D) Cre-/TMX- samples expressed the 354bp intact GPR54 product in all tissues and no band for the 800bp product in any tissue. (D) Genomic DNA from C57Bl/6 wild-type mouse islets was also tested as a control (WTC / WTK). The intact primers produce a 289bp product in wild-type mice due to the lack of the inserted LoxP sequence and the knockout primers have no band.

To assess the level of GPR54 knockdown islets were isolated from β-cell GPR54-/- mice and from Cre+/TMX- mice. Islet RNA was extracted and cDNA synthesised. GPR54 mRNA expression was measured by qPCR and quantified using the ΔΔCT method relative to the housekeeping gene, β-actin. (E) The islets of β-cell GPR54-/- mice have a significantly reduced level of GPR54 mRNA compared to Cre+/TMX- mice, with a loss of approximately 70% of GPR54 mRNA. Mean±SEM. n=3 mice per treatment (measured in triplicate) with each treatment containing 100-200 islets; *$p<0.05$.

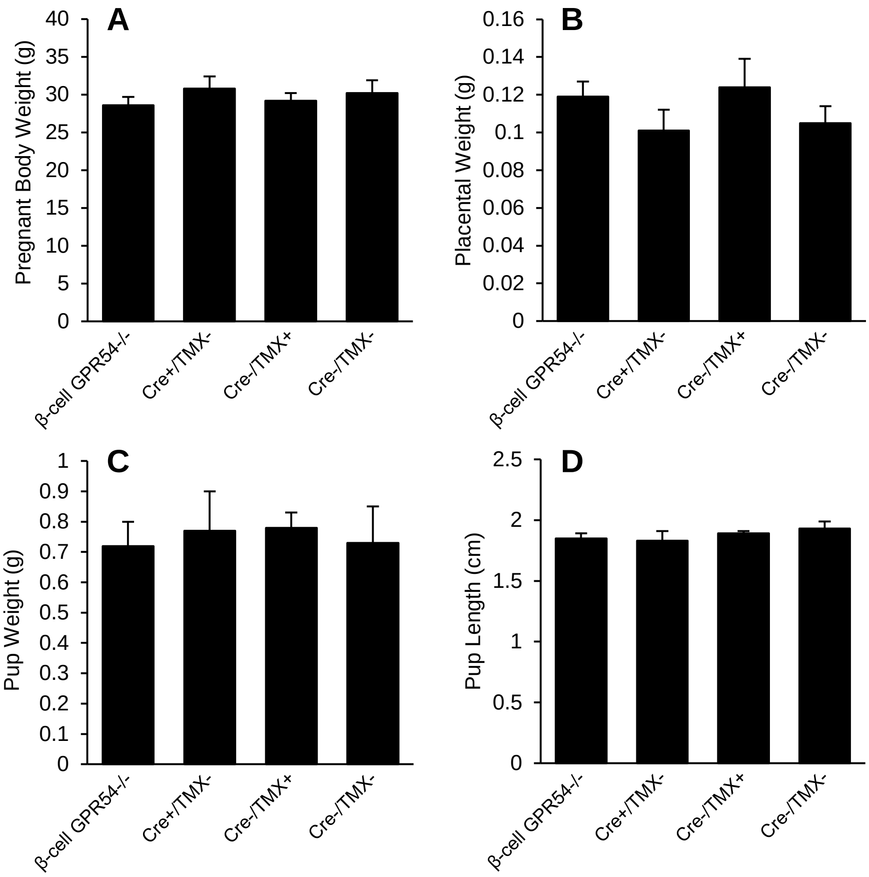

Figure S4: Characteristics of β-cell GPR54-/- pregnancy.

(A) There was no difference in gestational body weight at day 16 of pregnancy between β-cell GPR54-/- mice and any control group. Similarly, despite glucose intolerance, there was no significant difference in (B) placenta weight, (C) pup weight or (D) pup length between litters from β-cell GPR54-/- mice and any control group at day 18 of pregnancy.

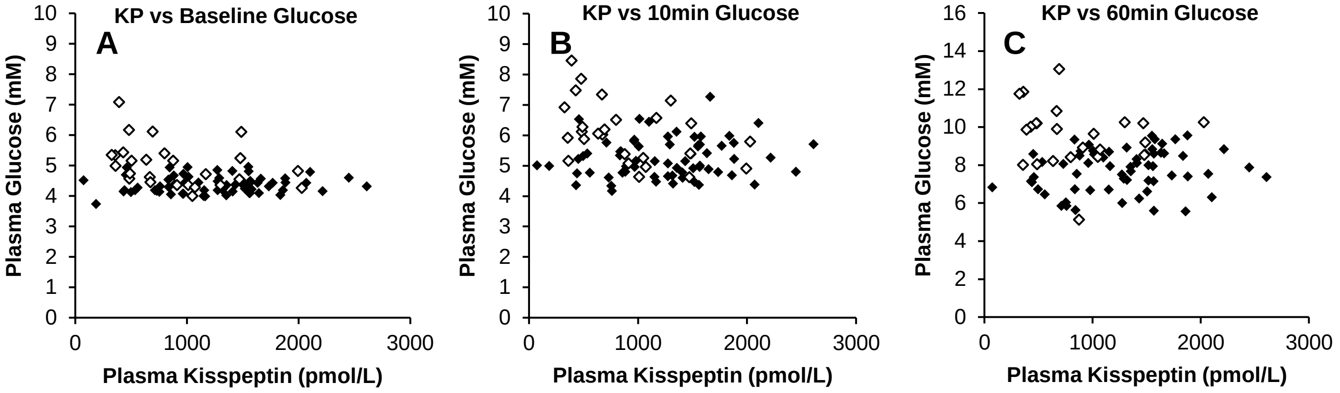

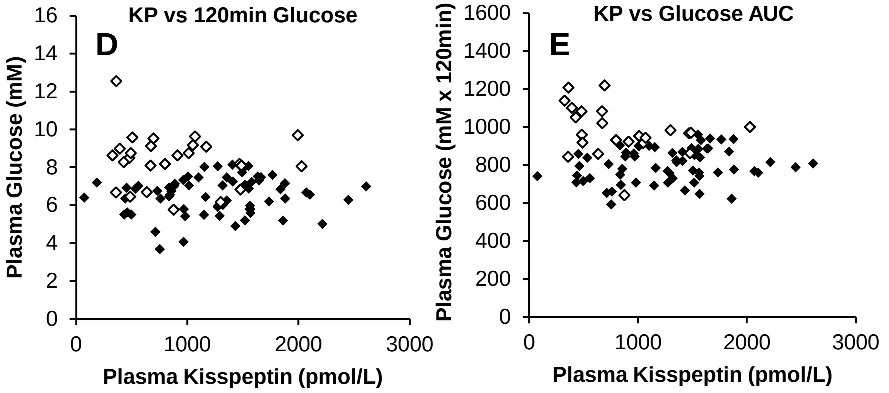

F

	Pearson's R correlation coefficient	*p* values
KP vs Glucose 0	-0.252	0.016*
KP vs Glucose 10	-0.197	0.061
KP vs Glucose 60	-0.122	0.266
KP vs Glucose 120	-0.154	0.144
KP vs Glucose AUC	-0.185	0.090

Figure S5: Relationships between kisspeptin and plasma glucose during an OGTT in pregnant women.

In pregnant women undergoing a routine 75g OGTT there was a significant negative correlation between kisspeptin and fasting plasma glucose (A, F). There was no correlation between kisspeptin and plasma glucose at 10, 60 or 120 min post glucose challenge (B-D, F) or between kisspeptin and AUC plasma glucose over the OGTT (E-F). Black symbols represent non-GDM women; white symbols represent GDM women.

	All participants	No GDM	GDM	No GDM vs GDM *p*-value	Test used
**Number of participants**	91	62	26		
**Age (Mean±SE)**	34.1±0.6	34.8±0.8	32.9±1.0	0.181	T test
**Ethnicity (number (%))**				0.817	Fisher's exact test
Black	23 (25.3%)	13 (21%)	7 (26.9%)		
South Asian	5 (5.5%)	3 (4.8%)	2 (7.7%)		
White	51 (56.0%)	37 (59.7%)	14 (53.8%)		
Other	12 (13.2%)	9 (14.5%)	3 (11.5%)		
**Singleton pregnancy**	88 (96.7%)	59 (95.2%)	26 (100%)	0.552	Fisher's exact test
**Gestation (median (range))**	29 (27-33)	29 (27-33)	29 (27-33)	0.654	Mann-Whitney U Test
**Number of previous pregnancies (median (range))**	1 (0-9)	1 (0-9)	2 (0-6)	0.204	Mann-Whitney U Test
**BMI**	30.5±0.7	29.6±0.8	32.3±1.2	0.069	T test
**Systolic blood pressure (mmHg)**	108±0.9	107±1.1	110±1.7	0.117	T test
**Diastolic blood pressure (mmHg)**	65±0.909	65.4±1.1	65.8±1.8	0.866	T test
**Kisspeptin (pmol/L)**	1146.8±57.1	1270.9±67.1	889.9±96.6	0.002	T test

Supplementary table: Characteristics of the pregnant women.

Of the 91 participants, 26 had GDM, 62 did not have GDM and 3 could not be classified as no GDM due to missing 60 min sample. There was no difference in age, ethnicity, multiple pregnancy, gestation, number of previous pregnancies, BMI, systolic blood pressure or diastolic blood pressure between women with GDM and women without GDM. Data is presented as Mean±SEM unless otherwise stated. $p<0.05$
